# Robust Drivable Road Region Detection for Fixed-Route Autonomous Vehicles Using Map-Fusion Images

**DOI:** 10.3390/s18124158

**Published:** 2018-11-27

**Authors:** Yichao Cai, Dachuan Li, Xiao Zhou, Xingang Mou

**Affiliations:** 1 School of Mechanical and Electronic Engineering, Wuhan University of Technology, Wuhan 430070, China; easoncai@whut.edu.cn (Y.C.); sunnymou@whut.edu.cn (X.M.); 2 California PATH, University of California, Berkeley, Richmond, CA 94804-2468, USA; dachuanli@berkeley.edu

**Keywords:** drivable road region detection, autonomous vehicles, map-fusion image, FCNs

## Abstract

Environment perception is one of the major issues in autonomous driving systems. In particular, effective and robust drivable road region detection still remains a challenge to be addressed for autonomous vehicles in multi-lane roads, intersections and unstructured road environments. In this paper, a computer vision and neural networks-based drivable road region detection approach is proposed for fixed-route autonomous vehicles (e.g., shuttles, buses and other vehicles operating on fixed routes), using a vehicle-mounted camera, route map and real-time vehicle location. The key idea of the proposed approach is to fuse an image with its corresponding local route map to obtain the map-fusion image (MFI) where the information of the image and route map act as complementary to each other. The information of the image can be utilized in road regions with rich features, while local route map acts as critical heuristics that enable robust drivable road region detection in areas without clear lane marking or borders. A neural network model constructed upon the Convolutional Neural Networks (CNNs), namely FCN-VGG16, is utilized to extract the drivable road region from the fused MFI. The proposed approach is validated using real-world driving scenario videos captured by an industrial camera mounted on a testing vehicle. Experiments demonstrate that the proposed approach outperforms the conventional approach which uses non-fused images in terms of detection accuracy and robustness, and it achieves desirable robustness against undesirable illumination conditions and pavement appearance, as well as projection and map-fusion errors.

## 1. Introduction

Environment perception is a critical technical issue for autonomous vehicles and significant progress has been made during the past decade. In particular, road detection is one of the key aspects of environment perception, since identifying key elements of the road (such as road markings and lane borders) and drivable road regions is essential for providing valid road information to support decision-making and to navigate the vehicle on roads [1].

In the area of autonomous driving systems, various environment perception systems have been designed based on different perceptions sensors (monocular/stereo camera, LiDAR and radar, etc.), among which LiDAR and camera are the most commonly used sensors for environment perception. Although LiDAR has been applied to perception tasks in several autonomous vehicle systems and demonstrated its effectiveness in experimental tests [2,3], its application in drivable road detection is still limited by its disadvantages such as high cost, low resolution and lack of texture information. Cameras are preferable to LiDAR in road detection due to their low cost, light weight, high resolution and simplicity in installation and configuration.

For camera-based perception systems, the objective of drivable road region detection is to identify a region of pixels that indicates the drivable (or navigation-free) area in a given image captured by the camera, and examples of applications of camera in road/lane detection can be found in [4,5,6,7,8,9]. The real-world autonomous driving scenarios pose significant challenges to camera-based perception systems, due to the following factors: (1) Unstructured road environments: road markings and lane borders are not always available, and the marking/borders may be too vague to be identified; (2) variable illumination conditions: the images may contain shadows and other undesirable illumination conditions; (3) road curvatures: the camera’s field-of-view may not capture the entire region needed due to curved road segments; (4) ununiform pavement appearance and occlusions: the road pavement in the image may contain variable texture and color, and the objects in the camera’s field-of-view may cause occlusions in the image. Despite the progress made in the road detection systems, the robust and effective detection of drivable regions in variable and challenging road environments remains a major challenge to be addressed.

To address the above issues for autonomous vehicles, this paper proposes a drivable road region detection approach using a monocular vehicle-mounted camera. This research focuses particularly on autonomous vehicles that have access to route maps (e.g., shuttles, buses and other vehicles operate on predefined fixed routes). Taking advantage of the route map and the vehicle location information, the proposed approach extracts from the full route map a local map which corresponds to the current processed image captured by the camera, which is then fused with the projected image to obtain a map-fusion image (MFI). The MFIs enable robust drivable region detection in the presence of undesirable illumination conditions, ununiformed pavement appearance, occlusions and absence of road/lane marking. In addition, for multi-lane scenarios, the lane navigation information (the lane which the vehicle is supposed to operate in) can also be provided in the MFI. The road detection approach is built upon the Fully Convolutional Network (FCN)-VGG16 framework which is trained using the MFIs. Therefore, the drivable road regions in each MFI are identified using the trained FCN-VGG16. The proposed approach is validated using video data collected from real-world scenarios, and experimental results show that the proposed approach achieves high accuracy and robustness in drivable road region detection in various driving scenarios and outperforms contemporary approach.

The remainder of this paper is organized as follows: Section 2 reviews the existing approaches for road detection. Section 3 formulates the drivable road detection problem. Details of the proposed road detection approach is presented in Section 4. Experimental results based on real-world scenarios are presented and analyzed in Section 5, and the paper is finally concluded in Section 6.

## 2. Related Works

In general, drivable road region detection can be classified as a road detection problem. During the past decade, various computer vision-based approaches have been proposed to address road detection problem for autonomous vehicle systems, and they can be generally divided into three categories. One straightforward paradigm detects the road regions by extracting the lane markings and road boundaries. This category of approaches utilizes conventional image processing techniques to extract the road boundaries by using image features such as edge, position and color [4,5,6,7,8,9]. After that, the boundaries are fitted (linear models [6,7] and high-order curves/splines are used for straight and curved road segments [8], respectively), and the area between the identified lane markings and/or boundaries is considered as the drivable road region. The conventional approaches have the primary advantage of offering the opportunity to evaluate specific performance criteria and factors that affect the performance, such as descriptors used. However, these approaches are sensitive to illumination variation and occlusion, and their performance may degrade significantly when dealing with roads with vague or discontinuous lane markings. Another category of approaches detects roads by analyzing texture features and attempting to find the inhomogeneity between road area and backgrounds in the image [10,11,12,13,14]. To specify texture information in the image, Gabor Filter [10,13] and discrete cosine transform [14] are commonly used in this type of road detection approaches and they can achieve desirable performance. Although this type of approaches performs well on rural, single-lane roads, their performance is undesirable when detecting drivable region on multi-lane roads. In general, the conventional approaches described above achieve desirable road detection performance only in specific scenarios and are less effective in more complex driving environments. For instance, the lane on the opposite direction of a two-way road may be incorrectly identified as drivable road region by the marking/boundary-based road detectors. In addition, there are usually multiple driving directions in intersection regions and it is difficult to identify correct drivable road regions only from boundaries and texture information. Therefore, the application of the conventional marking/boundary-based and texture-based approaches to real-world driving scenarios are still very limited due to their limitations.

As alternatives to the conventional approaches, machine-learning based semantic segmentation approaches have emerged as effective tools [15] in computer vision area, and due to their high precision and efficiency, they have been applied to a number of autonomous driving systems [16,17,18,19] in recent years. Semantic segmentation approaches can achieve pixel-level precision and demonstrate effectiveness in many road detection applications, and typical semantic segmentation algorithms are built upon CNNs, as proposed in [20,21,22,23,24]. A more recent example of such approaches is the Vanishing Point Guided Network (VPG-Net) [23] which can detect road under complex environmental condition. However, such CNN-based models typically process images using a local block as the input, which requires high computational cost. The performance of machine-learning-based approach are heavily affected by the dataset used to train the network and the size of required dataset may increase dramatically if the network is required to adapt to various use cases. In order to improve the efficiency of CNNs, the Fully Convolutional Networks (FCNs) is proposed in [25], which can recover the class of each pixel from abstracted features in the image. Since it is proposed, a number of research works have focused on making extensions and improvements to FCNs and applying the approach in the field of autonomous driving systems [26,27,28]. One of the most preferred variants is the MultiNet [28] which is a codec structure model based on FCNs and VGG [27], and it can accomplish joint classification, detection and semantic segmentation via a unified framework. The encoder of MultiNet is designed to extract the features from image, while the decoder is responsible for multiple tasks such as road detection. In the applications of road detection, the training parameters can be initialized with pre-trained VGGs to speed up the training speed, and the road region is detected using FCNs. MultiNet demonstrates appealing performance both in structured and unstructured road with a desirable trade-off between accuracy and training cost. Therefore, taking advantage of the machine-learning based frameworks, the approach proposed in this paper uses an FCN-VGG16 framework to identify drivable road region from map-fusion images.

On the other hand, real-world contains many complex driving scenarios that may be difficult to be handled by systems with single perception data source. As described previously, for intersections containing multiple lanes and multiple driving directions, it can be difficult to determine which direction the vehicle should be driving on by using solely the information of images. Therefore, fusing information from multiple sources is a preferable choice to improve the performance of road detection. [29] proposes a lane marking detection framework that fuses GPS data, vehicle velocity and lane map, and it demonstrates good performance in structured road scenarios. Similarly, GPS data and image are fused for localization and decision making in [30]. Inspired by the previous research, the vehicle location data and route map data are used to generate local route maps in the proposed framework. The vehicle location data can either be obtained from vehicle-mounted GPS or other data feeds such as point cloud map-matching of LiDAR data. We fuse a local route map and its corresponding image captured by vehicle-mounted camera into a map-fusion image (MFI), which provides high level heuristics for the drivable region detection. 

## 3. Problem Formulation

**Figure 1:**
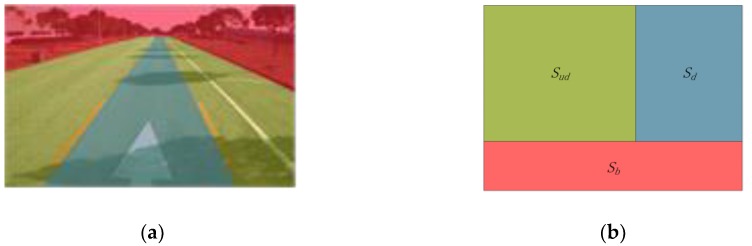
Definition of drivable region detection: (**a**) Regions in an image captured by the camera; (**b**) Relationship between the regions.

Drivable road region detection can be considered as a subtask of road detection which involves all key elements in the road environment. Without considering the pedestrians, vehicles and other obstacles, the drivable road region can be defined as the specific road area that the vehicles are supposed to drive in. For the monocular camera-based systems, the data precepted from the driving environment are the images captured by the vehicle-mounted camera. As shown in Figure 1a, a typical image of the driving environment can be divided into three parts: the background area, the drivable area and the non-drivable road area. Given an image *I* (Figure 1a) captured by the camera at certain timestamp t, the definitions used in drivable road region detection are as follows:

*S*—the set of all pixels in the image I, S={p|p∈I}
$S_{b}$—the set of pixels in the background area (or non-road area)
$S_{r}$—the set of pixels in the road region
$S_{d}$—the set of pixels in the drivable road region
$S_{ud}$—the set of pixels in the non-drivable road region.

The relationship between the above sets is as follows (Figure 1b):

(1) $$S=S_{b}\cup S_{r},$$

(2) $$S_{b}\cap S_{r}=\emptyset ,$$

(3) $$S_{r}=S_{d}\cup S_{ud},$$

(4) $$S_{d}\cap S_{ud}=\emptyset ,$$

Given the above definitions, the monocular camera-based road region detection problem can be formulated as follows:

**Definition** **1** **(Drivable** **Road** **Region** **Detection).** *Given a set of pixels S in an image I, extract the pixels of $S_{d}$ from S.*

In this paper, the objective of drivable road region detection is to extract the drivable regions in the captured images that correspond with the pre-specified route. In addition, for vehicles operating on fixed routes, note that the drivable road regions include regions of both single road segments and intersections.

## 4. Drivable Road Region Detection

**Figure 2:**
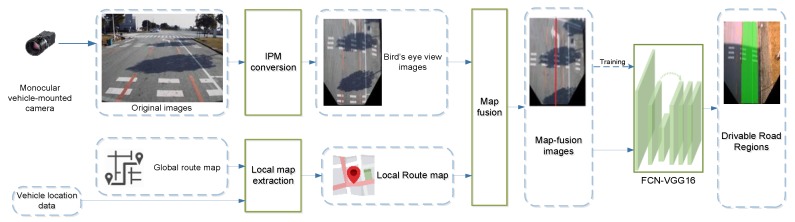
Overall Framework of the drivable road region detection based on map-fusion and monocular camera.

The overall framework of the proposed approach is depicted in Figure 2. Our approach consists of three primary processes: (1) Image pre-processing: the original images captured by the camera are swapped into bird’s-eye view images using Inverse Perspective Mapping approach. (2) Map fusion: the homogenous local route map of the processed image is extracted from a global route map with the assistance of vehicle location data, and it is then fused with the image to generate a map-fusion image (MFI). (3) Drivable road region detection: a FCNs-based deep neural network (FCN-VGG16) is utilized to detect drivable road region from the MFIs. Note that the FCN-VGG16 are also trained using datasets of MFI images. Details of each process are presented in the following subsections.

### 4.1. Inverse Perspective Mapping

**Figure 3:**
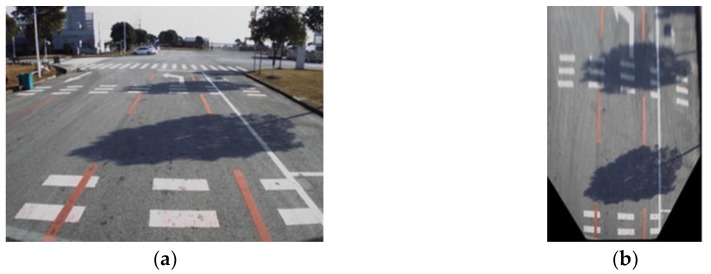
Images in front view and bird’s-eye view: (**a**) Front-view image; (**b**) Bird’s-eye-view image.

Vehicle-mounted cameras are typically installed in a way that the lens face toward the forward-driving direction of the vehicle. As depicted in Figure 3a, images captured by the vehicle-mounted camera are normally in the front-view, which is more suitable for certain detection applications such as pedestrian detection [31] and traffic-sign detection [32]. However, for drivable road region detection tasks, images in front-view contain a lot of redundant information which may affect the effectiveness of the detection algorithm. Compared with the front view, the bird’s-eye view is more preferable in drivable region detection applications as the image contains less redundant information, and the lane markings become roughly parallel [4] (Figure 3b). 

Without loss of generality, the road regions can be assumed to be homographic planes in road detection scenarios. Therefore, we can easily map the original images into bird’s eye view using Inverse Perspective Mapping (IPM). The IPM can be considered as an image projection process and in our application, the pixels in the original images can be projected to the bird’s eye-view images using a mapping matrix (projection matrix). In our application, a multiple-point correspondence-based method is used to estimate the mapping matrix $\hat{H}$:

(5) $$p_{i}=Hp_{i}^{\prime },\;(i=1,\;2,\;\ldots n)$$

(6) $$p=\hat{H}p\prime ,$$

where $p^{\prime }$ and *p* represent the homogeneous coordinates of pixels in the original image $I^{\prime }$ ($p^{\prime }\in I^{\prime }$) and the projected image I (bird’s-eye view) (p∈I), respectively. The mapping matrix $\hat{H}$ can be estimated in two-steps: First, select two sets of pixels ${p_{i}}^{\prime }$ and *p_i_* (*i* = 1, 2, …, *n*) from $I^{\prime }$ and I, respectively, and the optimal estimate ($\hat{H}$) of the mapping matrix H can be calculated based-on Equation (5). Once $\hat{H}$ is determined, all pixels of the front-view image $I^{\prime }$ can be projected into a bird’s-eye view image I using the estimated $\hat{H}$ (Equation (6)). (Note that the IPM is used in our approach to reduce redundant information instead of trying to ensure the lane markings are parallel.)

Note that the above described estimation of projection matrix $\hat{H}$ is a pre-calibration procedure, and the IPM process in our system uses the fixed $\hat{H}$ calculated via the pre-calibration. As a matter of fact, the roll and pitch movements of the vehicle can cause variations of $\hat{H}$, and therefore, a more accurate way of IPM is to adjust $\hat{H}$ based on the measurement of the vehicle’s roll and pitch in real-time. However, considering that the vehicle performs minor roll and pitch movements at low speeds in regular driving environments, the proposed drivable road detection approach can achieve robustness against such movements and small IPM error (see Section 5.4). Therefore, it is reasonable to use a fixed, pre-calibrated $\hat{H}$ in our application.

### 4.2. Map-Fusion Image Generation

**Figure 4:**
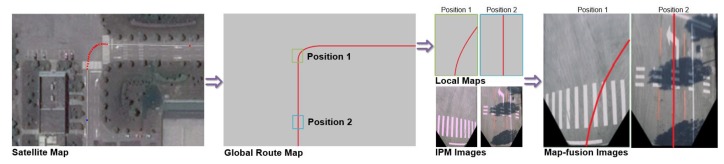
Generation of map-fusion images.

Figure 4 depicts the process of map-fusion image generation. The overall process consists of four steps which are described in the following subsections.

#### 4.2.1. Map-Fusion Image Generation

For vehicles driving on fixed routes (e.g., shuttles, buses and school buses) or vehicles with pre-established high-precision maps, the route maps provide critical heuristics for the environment perception and navigation of vehicles. A route map is defined as a map that consists of roads/lanes, navigation points and driving directions of the routes that the vehicle is supposed to operate on. In our system, the route map is presented and stored in the form of straight and curved segments (Figure 4), and each point in the route map represents a certain geographical location of the vehicle in the corresponding segment. In addition, for fixed routes, there is only one forward driving direction for the vehicle in a straight road segment or intersection, and this driving direction information is also included in the route map. The global map can be generated using various data sources such as GPS maps and high-precision 2D maps. For the framework proposed in this paper, a satellite map with geo-location information (i.e., GPS coordinates) is used to generate the global route map (Figure 4).

#### 4.2.2. Local Map Extraction

When the vehicle operates on the route which is pre-specified in the global route map, a local route map that corresponds with the road region in the current image from the camera is extracted from the route map every time an image is captured by the vehicle-mounted camera. In the proposed framework, the matching between the local route map and images is based on GPS data from the vehicle-mounted GPS module. For each frame of image and GPS coordinates, we search its corresponding position and localize the corresponding region in the global route map using GPS coordinates. After that, a local route map with a look-ahead length of 20 m can be extracted from the global route map, using the current position of the vehicle (according to the installation position of the camera, the equivalent height of each bird’s-eye-view image is approximately 20 m above the road surface, leading to a look-ahead distance of 20 m). Note that vehicle location from other data feeds (e.g., LiDAR point cloud matching) can also be utilized in the proposed approach.

The synchronization of the images and GPS data is a major issue in local route map extraction, as the update rates of the vehicle-mounted camera and the GPS module may be different. In our approach, it is appropriate to use the GPS data of which the timestamp is closest to that of the image, under the assumption that the vehicle would not move a long distance within milliseconds.

#### 4.2.3. Map-Image Fusion

After the local map is extracted, the image is fused with the corresponding local route map to generate a map-fusion image (MFI), where the road image contains the primary information, and the local route data provide heuristic navigation information of the autonomous vehicle, including location, specified lane and driving direction. 

As depicted in Figure 4, in the map-fusion image, the original bird’s view image is overlaid with line or curved segment representing the pre-defined route of vehicle specified in the corresponding local route map (Figure 4). The midpoint of the bottom border of the image represent the position of the vehicle at the moment the image is captured. Therefore, the origin of the line/curvature in the local map is aligned with the midpoint of the bottom border of the image. Experiment results indicate that the color and width of the line have slight effect on the detection results. Therefore, in our application, we use green-colored (RGB: 0, 255, 0) lines with a width of 2 pixels to represent the route in the MFI for the subsequent drivable road region detection process. However, for visualization purposes, broad red lines are used to represent the route in Figure 4.

### 4.3. FCN-VGG16 for Drivable Road Region Detection

#### 4.3.1. Neural Network Model

**Figure 5:**
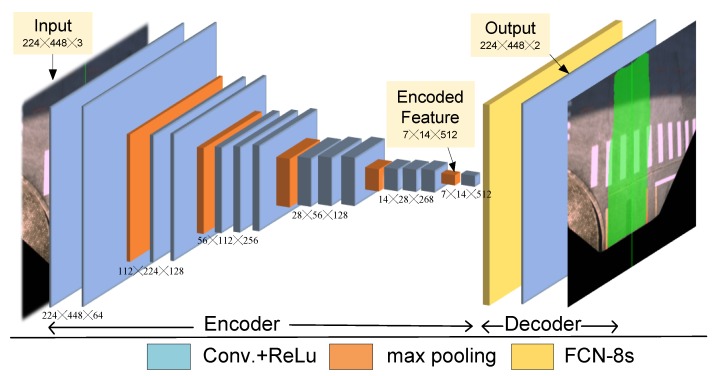
Segmentation model based on FCN-VGG16 [28].

As described in previous subsections, CNNs demonstrate desirable performance in various detection tasks due to its high efficiency and storage-saving characteristics. The neural network model used in this research follows an extension of the CNN model, namely the MultiNet, which is an end-to-end model firstly proposed in [28]. The MultiNet is a multi-task framework and the semantic segmentation model FCN-VGG16 of MultiNet is adopted in the proposed framework for drivable road region detection. The FCN-VGG16 model consists of two major components, a CNN encoder and a segmentation decoder. As an encoder in our framework, a pre-trained VGG16 network which consists of 13 convolutional layers is used to extract features from the MFI images. For the decoder module, the extracted features are processed by a cascade of convolutional layers and up-sampling layers to generate the output. A set of 1 × 1 convolutional layers is responsible for generating a low resolution segmentation. After that, three transposed convolution layers performs up-sampling of the low-resolution segmentation. Finally, skip layers composed by 1 × 1 convolutional layers extract high-resolution features and output the image with detected drivable road regions. The architecture of the FCN-VGG16 model is depicted in Figure 5.

#### 4.3.2. Selection of Loss Function

The Cross-entropy function is widely used as loss function in neural networks as it can effectively avoid the gradient vanishing problem. Therefore, loss function adopted in the training process of the FCN-VGG16 model is a cross-entropy function which is defined as follows:

(7) $$Loss\left(P,\;G\right)=\frac{1}{\left|I\right|}\sum_{i\in I}\left(G_{i}logP_{i}\right),$$

where I denotes the MFI inputs, |I| represents the size of the image, i represents a pixel in the MFI, $G_{i}$ is the ground truth of i, and $P_{i}$ is the predicted binary value of i. If i falls in the predicted drivable region, the value of $P_{i}$ is set to 1, otherwise $P_{i}$ equals to 0. $G_{i}$ is calculated in a similar way.

## 5. Experimental Study

### 5.1. Experiment Platform

**Figure 6:**
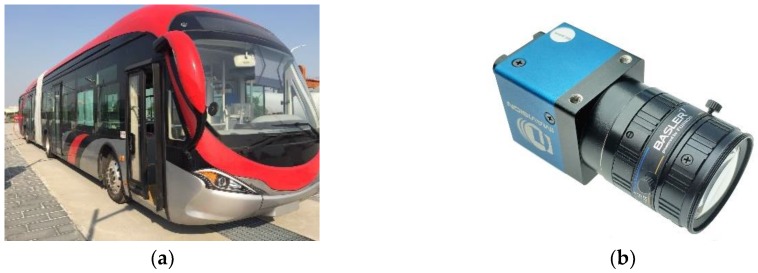
Experiment Platform: (**a**) 18 m testing bus, (**b**) vehicle-mounted camera.

The proposed map-fusion and FCN-VGG16-based drivable road region detection was tested using real-world driving scenario videos captured by a camera (Figure 6a) mounted on an 18-meter bus (Figure 6b). Parameters of the vehicle-mounted are listed in Table 1. To obtain a desirable field of view, the camera was installed down the center of the upper border of the windshield and below the headliner of the bus. Testing drive was conducted on a 4.3 km testing track which consists of 4 intersections, and videos of the testing drive and the corresponding GPS data were recorded to test the drivable road region detection algorithm.

To reduce the training time of the FCN-VGG16 model, we normalize the converted IPM images to a resolution of 224 × 448 (The original resolution is 1292 × 964, see Table 1). The experiments were conducted on a PC with one Intel^®^ Core i5-3570 CPU and one NVIDIA^®^ 1080Ti GPU. With a learning rate of 0.00001, it took 3.5 h to train the FCN-VGG16 network for 30000 times using the MFI images.

### 5.2. Evaluation Metric

The Pixel Accuracy (PA) [15] is used as the metric for evaluating the proposed approach. Since drivable road region detection can be regarded as a single-class semantic segmentation task, the calculation of PA can be simplified as follows:

(8) $$PA=\frac{p_{tp}}{p_{fp}+p_{tp}+p_{fn}},$$

where, $p_{tp}$, $p_{fp}$ and $p_{fn}$ represent the number pixels of true positive, false positive and false negative cases detected by the algorithm, respectively. Therefore, high Pixel Accuracy value indicate that the algorithm achieves good performance in detecting drivable road regions.

### 5.3. Evaluation of Detection Accuracy

A dataset of 270 frames is selected from the recorded video to evaluate the performance of the proposed approach. The dataset includes straight road segments, curved segments and intersections. For comparison purposes, the algorithm is tested using both map-fusion images (MFI) and raw images (without map-fusion). The PA of detection results are calculated for each frame in both cases.

**Figure 7:**
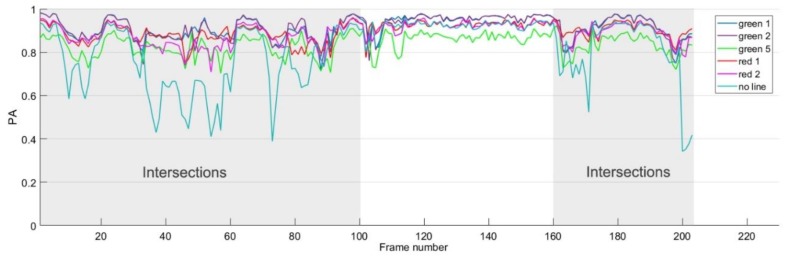
PA of detection for each frame.

The route line added to the MFI can be considered as artificial lane-markings. Therefore, the choice of color and width of the route line may affect the drivable road region detection results. The color of the route line should be distinct from the color of the road region in the image, and the width of the line is relevant to the intensity of the high-level intervention. We test different choices of colors and widths of the lines in local maps to demonstrate the effect of MFI in drivable road region detection. The performance (PA) of different choice of line colors and widths for each frame are depicted in Figure 7 (The cases using raw images without map-fusion are marked as ‘no line’).

As shown in Figure 6, the testing site contains two intersections and turning sections without lane markings, and they correspond with the first 100th frames, and the sections from the 160th to 203th frames of the dataset (the other sections correspond with regular straight road segments), respectively. As can be seen from Figure 7, the PA value of detection is quite low at intersections and turning segments (curve marked as ‘no line’) due to the absence of lane markings, and there is little information for the algorithm to determine the drivable road region. Therefore, the route line in the map-fusion image provides important heuristics for drivable road region detection.

As for the route line, experiment results demonstrate mild difference using different choices of line colors and width, as shown in Figure 6. Experiment results also suggest using colors that are distinctive from the color of road surface, such as green and red. In our experiment, we chose to use the green color and the width of 2 pixels for the route line in the MFI (please note that the route lines are rendered in red in the subsequent figures just for clarity of visualization purposes).

**Figure 8:**
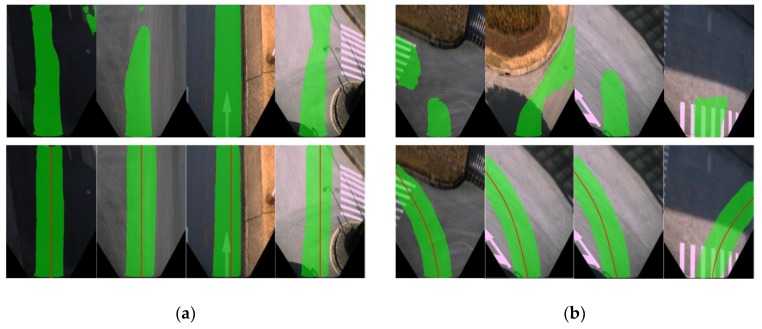
Comparison of detection results. The upper row shows the results of conventional FCN without MFI, and the lower row is the detection results of the proposed approach. (**a**) Detection results of straight road segments; (**b**) Detection results of intersections and turning segments.

By calculating the average PA value of all frames in the dataset, the average performance in detecting accuracy of FCN-based detection algorithm using raw images and our map-fusion-based algorithm is evaluated and listed in Table 2. The evaluation results indicate that the proposed approach outperforms conventional FCN-based detection algorithm in terms of detection accuracy (PA). Examples of detection results are depicted in Figure 8, which contains typical scenarios such as intersections, straight roads and turning sections, and the detected drivable road regions are marked in green. In contrast to conventional FCN-based detection algorithm, the proposed approach can still effectively detect drivable road detections even in intersections and turning segments where lane-markings are absent.

### 5.4. Evaluation of Robustness

The complex road scenarios of the testing site include absence of lane/road markings, illumination variation, disturbances in road surface appearance and the presence of various road users (other vehicles, pedestrians, bicycles and other road users). In addition, errors introduced by the IPM conversion and local route map extraction process may also lead to detection deviations. 

**Figure 9:**
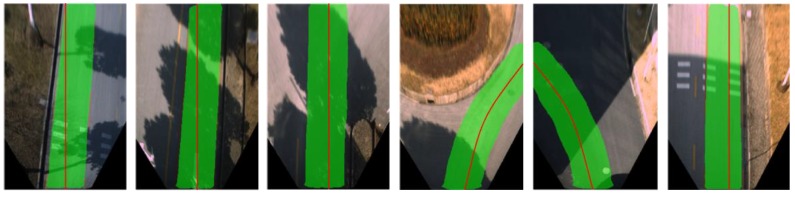
Robustness against illumination variation.

**Figure 10:**
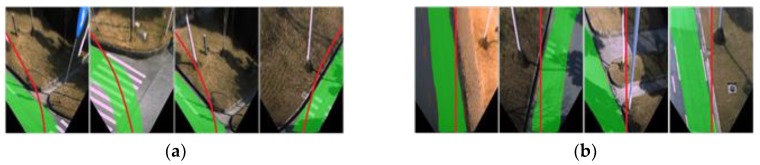
Robustness against conversion and fusion errors: (**a**) Detection results in the presence of local map extracting deviation; (**b**) Detection results in the presence of IPM errors.

**Figure 11:**
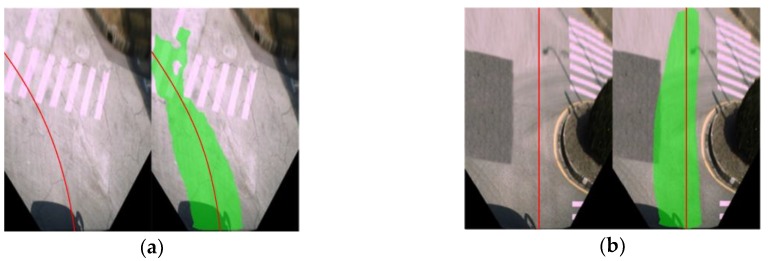
(**a**) Detection results in the presence of large cracks; (**b**) Detection results in the presence of inconsistent pavement colors.

The robustness of our approach was evaluated using the above complex road environments. Examples of the testing results are shown in Figure 9, Figure 10 and Figure 11. As described in Section 5.3, the proposed approach demonstrates desirable robustness in the absence of road/lane markings (Figure 8). Figure 9 depicts examples of typical illumination variation scenarios including heavy shadows and variations of illumination direction. As can be seen from the figure, the proposed approach achieves desirable robustness in such scenarios, while conventional border or feature-based approaches may fail.

Figure 10 demonstrates examples of incorrect local route maps (Figure 10a) and IPM conversions (Figure 10b) due to errors. As can be seen from the figures, the drivable road region can still be detected by our approach even when the route lines are placed in totally incorrect positions of the image. These results indicate that the combination of map-fusion and FCN-VGG16 can improve the robustness of drivable road detection in complex road environments. 

Figure 11 shows the detection results of the proposed approach in the presence of undesirable road surface appearance. Still, our approach demonstrates robustness against large pavement cracks (Figure 11a) and inconsistent pavement colors (Figure 11b).

**Figure 12:**
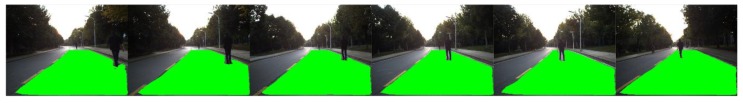
Detection results in the presence of single, continuously-moving pedestrian.

**Figure 13:**
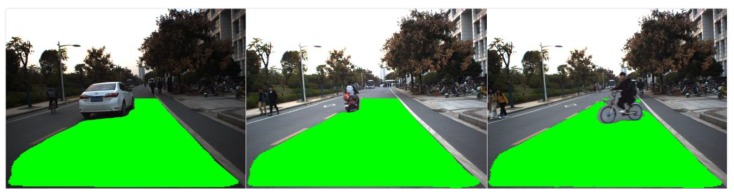
Detection results in the presence of various types of other road users.

**Figure 14:**
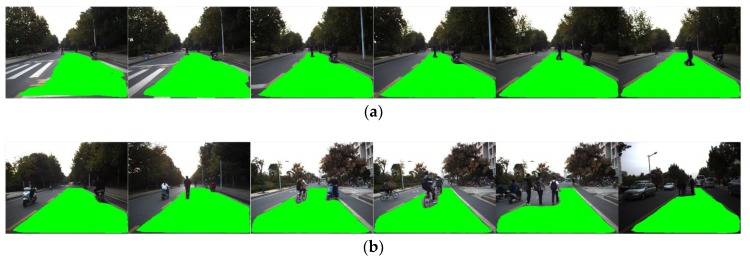
Detection results in the presence of: (**a**) Multiple road users; (**b**) various road users.

Figure 9 and Figure 10 present the performance of drivable road region detection in the presence of undesirable factors induced by illumination and road appearance. In most real-world driving scenarios, another aspect of the complexity of road environments is that the road region is usually shared by various road users such as other vehicles, pedestrians, and motorcycles, etc. We tested the propose drivable road region detection approach in various scenarios with other road users, as shown in Figure 12, Figure 13 and Figure 14 (for visualization purposes, these figures are shown in the front-view). Figure 12 presents the scenario with single, continuously moving pedestrian. The pedestrian walked across the lane the ego vehicle was driving in and away from the testing vehicle. As can be seen from Figure 12, the proposed approach can correctly and continuously extract drivable road regions as the distance between the pedestrian and the ego vehicle increased. 

Figure 13 shows detection results of various scenarios with different types of road users other than pedestrians (from left to right: other vehicle, motorcycle and bicycle). As can be seen from Figure 13, the road users are of different types and sizes, with different distances and orientation relative to the ego vehicle. As the FCN-VGG16 network was trained using image datasets containing various road users in different scenarios, the proposed approach can effectively identify drivable road regions when different types of road users share the lane the ego vehicle was driving in. 

Testing results in the presence of multiple and various road uses are shown in Figure 14. Figure 14a shows the scenario with a pedestrian and a motorcycle in the lane of the ego vehicle. The pedestrian walked across the lane and toward the ego vehicle, while the motorcycle drove in the same direction as the ego vehicle did with changing distances. Similar to the case shown in Figure 12, the proposed approach can continuously identify drivable road region as the two road users moved in the view of the camera. Figure 14b include more complex, multiple-road users driving scenarios that are common in real-world road environments. In such scenarios, the lane can be shared by multiple road users of different types, and they may drive in different directions. In addition, the opposite lane is usually occupied by other road users at the same time. As can be seen from Figure 14b, our approach can effectively deal with such scenarios and the testing results indicate that the proposed approach has the potential to be applied to autonomous vehicle systems operating in real-world driving scenarios. 

## 6. Conclusions

This paper proposes a robust drivable region detection approach based on a monocular vehicle-mounted camera for autonomous vehicles. The proposed approach takes advantage of the heuristics from the route map and vehicle location data and fuses the local route map to generate map-fusion images to improve the robustness of detection. The map-fusion images provide significant heuristics (e.g., heading, location, specified driving direction and lane of the vehicle) for drivable road region detection in complex road environments such as turning segments and intersections in the absence of road/lane markings. In order to extract drivable road regions, the map-fusion images are segmented using an FCN-based neural network which is trained also using a dataset consist of selected map-fusion images. The proposed approach is validated using real world videos captured by a camera mounted on a testing bus. Experiment results show that the proposed approach achieves desirable detection accuracy using an industrial camera, and it demonstrates robustness in complex driving scenarios involving absent road/lane markings, undesirable illumination conditions and pavement appearance, as well as various road users. Evaluation results also indicate that the proposed approach outperforms conventional FCN-based algorithm using raw images in terms of detection accuracy.

The future work will focus on the incorporation of other vehicle location data feeds such as LiDAR point-cloud matching. More comprehensive investigation is also required to further improve robustness of the proposed drivable road region detection approach. The implementation and integration of the drivable-road detection approach to an autonomous driving system will be investigated. In addition, more testing will be conducted to further evaluate the performance of the proposed approach in more real-world driving scenarios.

## Figures and Tables

**Table 1:** Parameters of the vehicle-mounted camera.

	Parameters
Model	Daheng MER-125-30-UC
CCD sensor	Sony^®^ ICX445
Resolution	1292 × 964
Frame rate	30 fps
Lens	Basler 4 mm fixed focus length

**Table 2:** Average detection accuracy.

Approach	PA
FCN without MFI	0.811
MFI and FCN-VGG16	0.917
